# Tuberculous peritonitis; The effectiveness of diagnostic laparoscopy and the perioperative infectious prevention: A case report

**DOI:** 10.1016/j.ijscr.2020.06.046

**Published:** 2020-06-13

**Authors:** Leo Yamada, Motonobu Saito, Tetsuro Aita, AungKyiThar Min, Eisei Endo, Koji Kase, Daisuke Ujiie, Hiroyuki Hanayama, Hirokazu Okayama, Wataru Sakamoto, Hisahito Endo, Shotaro Fujita, Zenichiro Saze, Tomoyuki Momma, Shinji Ohki, Sugihiro Hamaguchi, Koji Kono

**Affiliations:** a Department of Gastrointestinal Tract Surgery, Fukushima Medical University, Japan; b Department of General Internal Medicine, Fukushima Medical University, Fukushima, Japan

**Keywords:** Tuberculous peritonitis (TBP), Diagnostic laparoscopy, Perioperative infection control, Minimal invasion, Laparoscopic features

## Abstract

**Background:**

Tuberculous peritonitis (TBP) is uncommon in Japan, and its diagnosis with conventional methods is time taking and requires a high clinical index of suspicion. Laparoscopy with peritoneal biopsy is a tool for rapid and accurate diagnosis of TBP. However, few cases have mentioned the infectious control and prevention during the perioperative period.

This case is written following the SCARE scale for case report writing.

**Case presentation:**

A 30-year-old man from Southeast Asia with a past medical history of pulmonary tuberculosis at 3-year-old admitted to our institution with abdominal pain and slight fever lasting for a week. With the elevation of inflammatory response and CA125, we conducted CT (Computed tomography). Not only ascites, panniculitis with peritoneal nodules, and the thickening of the omentum were found. Considering the possibility of malignancy and TBP, we performed a diagnostic laparoscopy. Slightly cloudy ascites, peritoneal and thickening omentum with white nodules were seen, and pathological diagnosis from the omentum during the operation raised the possibility of TBP due to its caseating granuloma and these findings allowed us to start the rapid treatment.

**Conclusion:**

We reported the effectiveness of diagnostic laparoscopy along with the aspect of perioperative prevention for TBP.

## Highlights

• A rare case of Tuberculosis Peritonitis regarding the aspect of perioperative infection control.
• The importance of characteristic intraperitoneal findings, which contributes to the rapid and accurate diagnosis combined with pathological findings.
• Diagnostic laparoscopy must be less invasive and more effective compared with conventional methods.

## 1 Background

In Japan, compared with other Asian countries, the number of tuberculosis (TB) patients is under control, and it is relatively difficult to find tuberculosis peritonitis (TBP).

TBP accounts for only 0.04–0.7% of all tuberculosis, and the nonspecific clinical symptoms and features often delay the early treatment [1,2].

But the number of patients diagnosed with TBP has been increasing in parts of the world where TB was rare partly due to the increasing travel and migration and also to the rising number of immunodeﬁciency disease [3]. So, we should list in the differential diagnosis for abdominal disease, which has nonspecific clinical symptoms, unidentified ascites, and peritonitis.

Compared with conventional methods, laparoscopy is a more accurate and effective approach to make a rapid diagnosis and treatment for TBP. The characteristic intraperitoneal features such as white nodule, thicken omentum are high sensitivity (93%) and specificity (98%) combined with the histological findings [3].

But at the same time, we have to consider the infectious prevention during the perioperative period.

This work has been reported in line with the SCARE criteria [18].

## 2 Case presentation

A 30-year-old man from Southeast Asia presented with right lower abdominal pain, fatigue, and slight fever lasting for a week. As a past medical history, pulmonary tuberculosis was treated with the combined use of 4 kinds of anti-tuberculous drugs (isoniazide, rifampicin, ethambutol, and pyrazinamide) for half a year when he was 3-years old. There were no respiratory symptoms when he was admitted to our hospital.

**Fig. 1:**
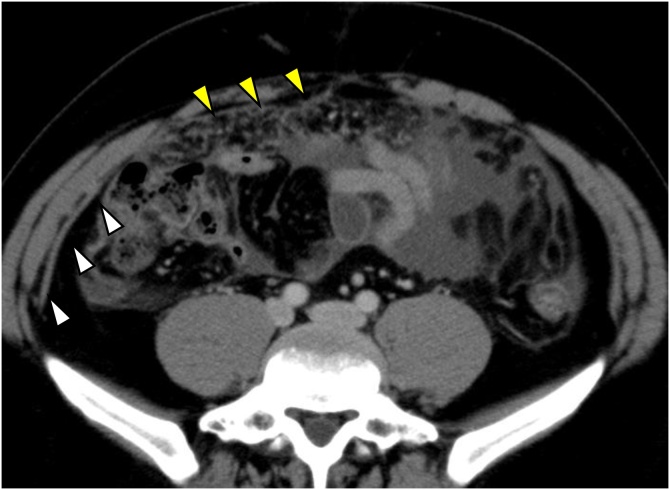
Chest-abdominal computed tomography showing ascites fluid, and panniculitis with peritoneal nodules (white arrows) and the thicken of the omentumn(yellow allows).

High score of CRP (13 mg/dl) and CA125 (486 U/mL) was pointed out by blood test, and ascites fluid was found by abdominal ultrasound. Chest-abdominal CT scan revealed no sign of pulmonary tuberculosis, but massive ascites and panniculitis with peritoneal nodules and the thickening of the omentumn (Fig. 1).

The possibility of tuberculosis peritonitis and malignancy could not rule out, and we performed a diagnostic laparoscopy three days after admission to the TB ward.

Before the operation, in the aspect of infection control and prevention, we carried out the direct smear examination three times, which were all negative and prepared N95 masks, Goggles, and Negative pressure room during the operation following the standard precaution for pulmonary tuberculosis.

**Fig. 2:**
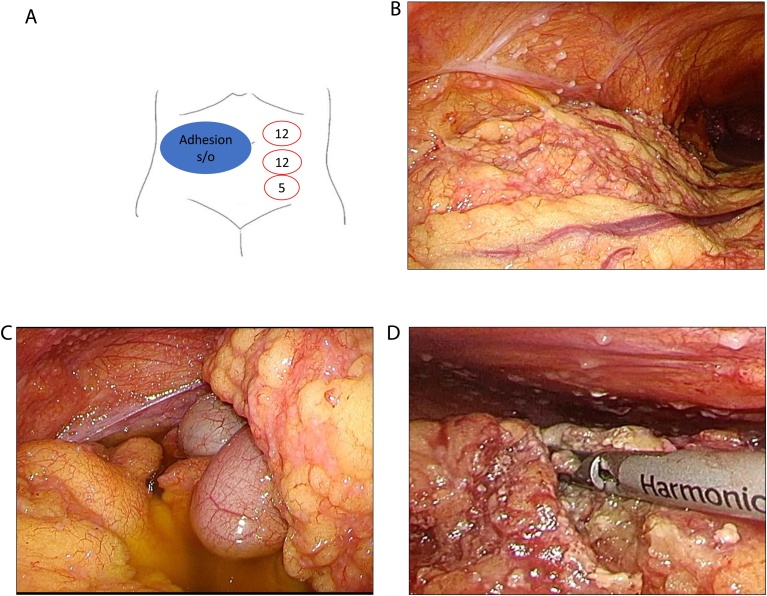
Scheme of ports position attempted to avoid the adhesion area (a). Numerous white nodules (a few millimeters) at the abdominal wall and omentum (b). Ascites fluid with slightly cloudy at the both paracolic and thicken omentum with white nodules (c). Obtaining historical specimens from peritoneum and omentumn using Ultrasonic incision coagulating device (d).

With the patient in a supine position, we used three ports (Fig. 2A 12 mm for scope, 12 mm for Harmonic and 5 mm) and rolled the port position on the left side of hilum because adhesion around the hilum was expected. Intraperitoneal findings were very characteristic; 1. Ascites with slightly cloudy at the both paracolic sulcus and rectovesical pouch, 2. Numerous white nodules (a few millimeters) at the abdominal wall, 3. Thicken omentum with white nodules on the surface (Fig. 2B, C).

During the operation, we performed biopsy from peritoneum and omentum for rapid histological evaluation and pathological diagnosis (Fig. 2D). In addition, the culture and PCR using peritoneum, omentum, and the ascitic fluid were carried out. PCR with peritoneum and omentum found out to be negative 7 days after operation. Acid-fast bacilli (AFB) culture with peritoneum and omentum tested positive of *Mycobacterium tuberculosis* 25 days after operation.

**Fig. 3:**
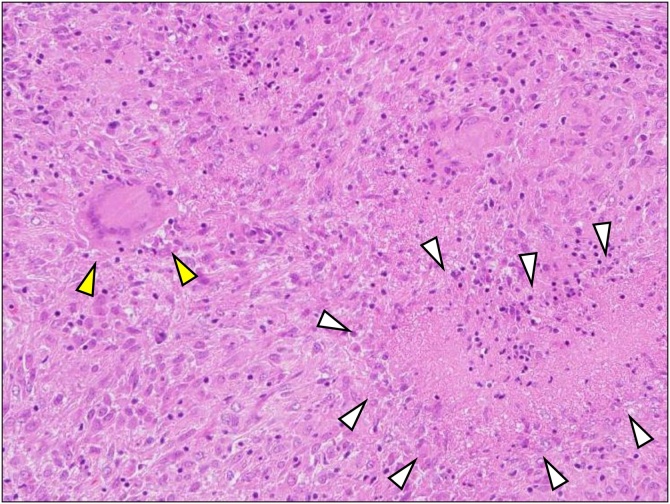
Microscopic findings of peritoneal biopsy specimens (H&E). Chronic granulomatous inflammation with central necrosis (white arrow) and Langhans giant cell (yellow arrow) were seen, which suggested tuberculous peritonitis (×200).

But Langhans giant cell and caseating granuloma with necrosis was confirmed by the rapid pathological examination from the peritoneum (Fig. 3), and the score of adenosine deaminase (ADA) resulted in a high score (136.7 U/L) during the operation. Hence, we could start the anti-TB drugs on the next day of operation. No drain was placed due to the less invasive, and the postoperative course was uneventful. He discharged 6 days after surgery. Treatment with anti-TB drugs continued 6month, and after that, the patient is currently under observation.

## 3 Discussion

Tuberculous peritonitis (TBP) accounts for only 0.04–0.7% among all forms of TB worldwide and is relatively rare. Both sexes are equally affected, and the susceptible age is between 20’s and 40’s [[1], [2], [3]]. Infection of the TBP is usually secondary to haematogenous spread of tubercles from a pulmonary focus. However, coexistent active pulmonary disease is rare and occurs in up to 3.5% of pulmonary TB. The complication of intestinal obstruction is about 11–20%. Latent focus in the peritoneal cavity, might be activated by triggers (alcohol, liver disease, haemodialysis, HIV…) at an interval [[4], [5], [6]]. Treatment for TBP generally takes 6 months with the ﬁrst-line anti-tuberculous drugs (isoniazide, rifampicin, ethambutol, and pyrazinamide) in proportion to pulmonary tuberculosis [7].

Variable methods have been invested in studying TB, but concerning TBP, these experiments are often time taking or nonspecific. Only ADA(cut off: 32-36U/l) has high sensitivity (83.3–100%) and specificity (95–100%) [8]. Rest of the approaches are low rate of test positive and time taking such as ascitic fluid smear (3–10%), AFB culture (20–50%) and PCR (48%) [9,10]. Also, CA125 is referred to as a valuable marker due to the reflection of intraperitoneal inflammation [11,12]. CT (Computerized tomography) reveals characteristic features such as ascites, nodules (a few millimeters), thickening of peritoneum, and omentum, but it is difficult to rule out malignancy [13,14].

So, diagnostic laparoscopy might be one of the most reliable approaches for rapid and accurate diagnosis because of its high sensitivity (93%) and specificity (98%) combined with the histological findings [3]. It allows inspection of the intraperitoneal findings, and also offers the option to obtain specimens. There are three types of laparoscopic features:1; thickened, hyperaemic peritoneum with ascites and whitish miliary nodules (<5 mm) scattered over the parietal peritoneum, omentum and bowel loops (66%), 2; thicken and hyperaemic peritoneum with ascites and adhesions (21%), 3;markedly thickened parietal peritoneum with possibly yellowish nodules and cheesy material along with multiple thickened adhesions (fibro-adhesive type – 13%) [15].

Although it is difficult to obtain tissue for microbiological or histological assessment, these intraperitoneal findings are quite beneficial to initiate the treatment because some studies have consistently reported a specificity in excess of 96% on the laparoscopic appearance alone [3]. Besides, in the aspect of safety, complications of laparoscopy are rare (<3%: bleeding, infection, and bowel perforation), and the reported mortality is up to 0.04% [16].

It was thought that these findings were seemed to be sufficient for the diagnosis of TBP. Furthermore, because we considered that the possibility of malignancy was low by the negative fecal blood test result, we did not examine the tumor markers such as CA19-9 and CEA before performing a diagnostic laparoscopy. In fact, the diagnosis of TBP was made quickly by a diagnostic laparoscopy, and he could receive the treatment for TBP as early as possible.

Few papers refer to the infection control of TBP, but Paul A Jensen et al. mentioned in the Guideline for Preventing the Transmission of *Mycobacterium tuberculosis* in Health-Care Settings, only laryngeal and pulmonary tuberculosis are said to be contagious [17]. In this case, considering the intraperitoneal aerosol during the operation, although the laparoscopy system is a closed circuit, we prepared the N95 mask, Goggles, and Negative pressure room in proportion to pulmonary tuberculosis.

Usually, diagnostic laparoscopy will not take a long time, and it did not matter to accomplish with this equipment.

## 4 Conclusion

Unidentified ascites and peritonitis must be difficult for making diagnosis by conventional methods. Laparoscopy might be supportive of making a rapid diagnosis and start early treatment, although we should consider in the aspect of infectious prevention.

## Conflicts of interest

The authors declare that they have no conflict of interests.

## Funding

We have no disclosures of financial support relating to this study.

## Ethical approval

The study is exempt from ethnical approval in your institution please state this.

## Consent

The patient provided permission to publish the features of his case. The identity of this patient has been protected.

## Author contribution

Leo Yamada: performed surgery, conception of report, data collection, data analysis, manuscript writing, revision and submission.

Motonobu Saito: performed surgery, data analysis, manuscript writing and manuscript revision.

Wataru Sakamoto: performed surgery, data analysis, manuscript writing and manuscript revision.

Tetsuro Aita: suggesting differential diagnosis in terms of internal medicine, manuscript writing and manuscript revision.

Shinji Ohki: data analysis, manuscript writing and manuscript revision.

Koji Kono: data analysis, manuscript writing and manuscript revision.

All the other authors equally participated in the care of the patient. All authors participated in the acquisition, analysis, or interpretation of the data; drafting and revising of the manuscript; and the final approval of the paper. Furthermore, all authors agreed to be accountable for the integrity of the case report and have read and approved the final manuscript.

## Registration of research studies

NA.

## Guarantor

Dr Leo Yamada.

## Provenance and peer review

Not commissioned, externally peer-reviewed.
